# Factors Influencing Oral Health-Related Quality of Life in Older Adults in Rural Areas: Oral Dryness and Oral Health Knowledge and Behavior

**DOI:** 10.3390/ijerph18084295

**Published:** 2021-04-18

**Authors:** Eunju Choi, Dukyoo Jung

**Affiliations:** College of Nursing, Ewha Womans University, 52 Ewhayeodaegil Seodaemungu, Seoul 03760, Korea; celestial_@naver.com

**Keywords:** xerostomia, knowledge, behavior, quality of life, aged

## Abstract

The oral health of older adults is an important factor affecting their overall health and quality of life. This study aimed to identify the characteristics of oral health of older adults living at home in rural areas and investigate factors affecting oral health-related quality of life (OHRQoL), especially oral dryness and oral health knowledge and behavior. A descriptive correlational study was conducted. In total, 117 participants were included in the final analysis. Data were collected using questionnaires for oral dryness, oral health knowledge and behavior, and OHRQoL. In addition, oral dryness was measured by mechanical measurements. Oral health knowledge was positively correlated with oral health behavior (r = 0.18, *p* = 0.029) and OHRQoL (r = 0.25, *p* = 0.003). In addition, a positive correlation between oral health behavior and OHRQoL (r = 0.24, *p* = 0.005) was observed. Multiple regression analysis revealed that subjective oral dryness (β = −4.99, *p* = 0.001) had a significant effect on OHRQoL. To improve OHRQoL in the rural elderly, health providers should pay attention to oral dryness and comprehensively evaluate it. The development of prevention programs and continuous education that can improve oral health knowledge and behavior is also required.

## 1. Introduction

Oral health is a key indicator of overall health, well-being, and quality of life [1]. In particular, oral health is very important for older adults, as it is closely related to mortality and systemic diseases [2,3,4]. Moreover, oral health problems in older adults adversely affect their lives and diminish quality of life; thus, solving these problems is important.

One of the most common oral health problems in older adults is oral dryness, which affects one-third of older adults [5]. Oral dryness in older adults is also associated with decreased saliva secretion due to aging or drugs [6]. Oral dryness causes mastication and swallowing problems, as well as oral dysfunction such as pronunciation and taste issues, and increases the probability of tooth loss, dental caries, and periodontal disease [7,8], ultimately lowering oral health. Thus, oral dryness significantly affects the oral health-related quality of life (OHRQoL) [9].

Risk factors for oral health diseases include social and cultural factors [10]. One of these factors is living in rural areas as this leads to inequities in education and health and limits access to medical services [11]. In Korea, the number of medical institutions in rural areas is only 12.9% of that in urban areas [12]. In particular, there are 15,826 dental clinics in urban areas and 1788 in rural areas, the latter being only 11.3% of the former [13]. Therefore, rural residents have a high tendency to not receive adequate treatment in time, and the rate of dental screening is low [13]. Due to this, the role of public health institutions has become more important, but the function of the public sector has declined due to operational problems [14]. Moreover, relative to urban areas, rural areas have risk factors related to low income, low education, and high age [15].

To solve this regional disparity, the need for a preventive health policy was emphasized [16]. In particular, most oral health problems in older adults can be easily prevented through routine oral healthcare [17], which further emphasizes the importance of such healthcare habits. To maintain oral health, proper oral health behavior and accurate oral health knowledge are essential [18]. Indeed, increased oral health knowledge gives an individual a sense of personal control over oral health behavior and improves oral health [19]. Moreover, previous studies investigating factors that influence OHRQoL have shown that oral health knowledge and behavior act as important predictors [20,21,22]. However, there are no studies evaluating these factors for older adults in rural areas in Korea.

Therefore, this study aimed to explore the level of oral dryness, oral health knowledge and behavior, and OHRQoL of older adults in rural areas in Korea, and identify factors significantly influence OHRQoL. This knowledge may help healthcare providers who are at the frontline of oral health promotion in rural healthcare, improve the oral health of rural older adults who are marginalized in terms of medical benefits, and seek ways to prevent oral health problems. This study also aimed to provide a basis for developing interventions that can improve OHRQoL in this population.

## 2. Materials and Methods

### 2.1. Design

This is a descriptive survey conducted to identify the factors influencing OHRQoL in older adults in rural areas. 

### 2.2. Participants

Participants were older adults aged over 65 years living in W County, Jeollabuk-do, South Korea. Inclusion criteria comprised older adults 65 years or older (1) living at home in rural areas, (2) who were able to communicate to answer the questionnaire, and (3) who were able to cooperate when measuring oral dryness. Older adults with cognitive impairments such as mental illness or dementia were excluded. We offered survey incentives in line with site policies: older adults who participated in the survey received a gift of about $5. The sample size was calculated using the G*Power 3.1.9.4 program [23]. Assuming an effect size of 0.15 [24], a statistical power of 0.80, and a significance level of 0.05 for a multiple regression analysis, the minimum required sample size was 114. Based on this, 130 participants were recruited, considering a dropout rate of 10%. In the final analysis, 117 were included, excluding omissions and dropouts.

### 2.3. Data Collection

Convenience sampling was used in this study. Data were collected from rural areas of W county, Jeollabuk-do from 5 April to 20 May 2020. In this study, rural areas are defined as towns and villages where residents mainly engage in agriculture [25,26]. In W county, more than 31% of residents engage in agriculture and about 30.8% of the area is farmland [27]. W County comprises 555 villages in 3 towns and 10 townships. The advertisement flyers for recruiting study participants were distributed to the heads of villages in W County. The study was conducted with the elderly aged 65 years or above residing in 14 villages in rural areas of W-gun who agreed to participate in the study. Those who voluntarily wished to participate were asked to use the researcher’s contact information. The data collection was conducted in the resting place for farms and homes in rural areas. Due to the COVID-19 pandemic starting in December 2019, the COVID-19 prevention and control measures (wearing a mask, using hand sanitizer, etc.) were followed in all processes. After providing written consent for participation in the study, the completion of the questionnaire took about 15 min.

### 2.4. Ethical Considerations

Ethical approval for this study was obtained from the Institutional review board of Ewha Womans University in Korea (approval No. 202004-0008-02). The researcher explained the purpose, necessity, and anonymous nature of the study, and that participation could be withdrawn at any time and the contents of the survey would only be used for research purposes. Participants understood the purpose and necessity of the research and agreed to participate in advance. We ensured the protection of participants’ confidentiality.

### 2.5. Measurements

This study involved structured questionnaires consisting of 38 questions: 10 demographics and oral health related characteristics, 8 oral health knowledge measures, 6 oral health behavior measures, and 14 OHRQoL.

#### 2.5.1. Demographics and Oral Health Characteristics

The general characteristics included gender, age, educational level, marital status, monthly income level, and present illness. Oral health characteristics were investigated including oral health education experience, remaining teeth, and oral dryness. Oral dryness was measured in two ways: subjective and objective dry mouth.

The degree of objective oral dryness was measured using an oral moisture-checking device (Moisture^®^, approval number: 22200BZX00640000, Life Co., Ltd., Saitama, Japan). In a study by Fukushima et al. [28], the sensitivity and specificity of this device were 81%. The measured values reflect not only the water content of the oral mucosal surface, but also the intramucosal water content to a depth of approximately 50 μm. This measurement has been approved as a medical device for evaluating oral dryness and is widely used in the clinical field [28].

This device was used with a disposable sensor cover, and the mucous membrane moisture in the middle of the tongue mucosa, 10 mm from the tip of the tongue, was measured. To properly measure the oral mucosa moisture, the device was pressed against the tongue mucosa with a pressure of 200 g [28]. To minimize the abnormality caused by the angle of measurement of the sensor, a total of three measurements were taken, and the median value was used as the measurement value. The displayed value does not have a unit because it is a relative value. The higher the value, the higher the saliva secretion; values of 29.6 or higher indicate normal salivation, 28.0–29.5 indicate insufficient saliva secretion, and 27.9 or lower indicate oral dryness [29].

#### 2.5.2. Oral Health Knowledge

The oral health knowledge instrument in this study was modified and supplemented [30] based on an oral health knowledge instrument developed in a previous research [31]. The tool consists of eight items: three regarding tooth decay, one regarding brushing, one regarding oral examination, one regarding scaling, and two regarding oral care. For each question, “yes” was scored as 1 point, and “no” and “don’t know” were scored as 0 points; the higher the score, the higher the knowledge about oral health. Cronbach’s α was 0.61 in this study, as opposed to 0.70 in a previous study [30]. 

#### 2.5.3. Oral Health Behavior

The oral health behavior instrument [32] developed in previous studies was used to evaluate oral health behavior. It comprises six items (i.e., preventive dental visits, therapeutic dental visits, brushing education, brushing times, number of oral hygiene products used, and regular scaling) rated on a 5-point Likert-type scale from of 1 (never) to 5 (very often). The higher the score, the better the oral health behaviors were. Cronbach’s α was 0.76 in this study, as opposed to 0.62 in a previous study [32].

#### 2.5.4. OHRQoL

The adapted and shortened version of the Korean version of the Oral Health Impact Profile (OHIP-49) [33], the OHIP-14 [34], was used in our study. It has 14 items (i.e., two on functional restrictions, two on physical pain, two on psychological discomfort, two on lower physical ability, two on lower psychological ability, two on lower social ability, and two on social disadvantages), rated on a scale from 1 (never) to 5 (very often), resulting in a total score ranging from 14 to 70. Higher scores indicate a higher quality of life related to oral health. Cronbach’s α was 0.93 in this study, as opposed to 0.88 in a previous study [34].

### 2.6. Statistical Analysis

SPSS/WIN v22.0 (IBM Corporation, Armonk, NY, USA) was used for data analysis. Descriptive statistics (frequency, percentage, mean, and standard deviation) were used to summarize the data. Differences in oral health knowledge, oral health behavior, and OHRQoL according to general and oral health characteristics were analyzed by *t* test and one-way ANOVA with a Scheffe post-hoc test. The correlation between the participants’ oral health knowledge, oral health behavior, and OHRQoL was analyzed using Pearson’s correlation coefficient. The effect of variables on OHRQoL was analyzed using multiple linear regression. 

## 3. Results

### 3.1. Demographic and Oral Health Characteristics

The general and oral health characteristics of the study participants are shown in Table 1. The total number of study participants was 117, with 69 males (59%) and 48 females (41%). Participants’ ages ranged from 65 to 88 years, with a mean age of 69.85 ± 5.21 years. Regarding education, the highest proportion was that of high school graduates, with 35 participants (30%). The average monthly income of 700,000 won to 1.5 million won was the most with 45 participants (38.5%). A total of 103 participants (88%) had a chronic disease. As for the characteristics related to oral health, the number of participants without oral health education experience was 81 (69.2%), and the average number of remaining teeth was 18.81 ± 8.97. Regarding objective oral dryness, 112 (95.7%) had oral dryness; however, only 43 (36.8%) subjectively answered that their mouth was “dry.” Objective oral dryness was indicated as 27.9 or lower on the device. 

### 3.2. Oral Health Knowledge and Behavior, and OHRQoL

The mean score of oral health knowledge was 4.67 (SD 1.61) (range, 0–8). The oral health behavior score was 16.2 (SD 4.12) (range, 0–30), and the OHRQoL score was 42.40 (SD 12.18) (range, 14–70).

### 3.3. Oral Health Knowledge and Behavior, and OHRQoL, According to General and Oral Health Characteristics

Table 2 shows the differences in oral health knowledge and behavior and OHRQoL, according to general and oral health characteristics. There was no difference in oral health knowledge and behavior and quality of life, according to sex, chronic disease, or objective oral dryness. Oral health knowledge differed according to age (F = 3.815, *p* = 0.025). The oral health knowledge score of those aged 65–69 years was 4.96 (SD 1.47), which was higher than 4.07 (SD 1.70) in those aged 70–79 years. Oral health behavior differed based on age (F = 7.082, *p* = 0.001), education level (F = 10.663, *p* < 0.001), and average monthly income (F = 7.493, *p* = 0.001), with higher age, lower education, and income leading to worse oral health behaviors. OHRQoL differed according to age (F = 7.514, *p* = 0.001), education level (F = 7.013, *p* < 0.001), and average monthly income (F = 5.752, *p* = 0.006), with higher age, lower education, and lower income being associated with lower OHRQoL. There were no significant differences in oral health knowledge and behavior and OHRQoL, according to gender or present illness. 

There was no significant difference in oral health knowledge according to oral health characteristics. Oral health behavior differed according to the number of remaining teeth (F = 5.814, *p* = 0.004) and subjective oral dryness (F = 5.463, *p* = 0.005). Participants with fewer than 20 remaining teeth and those who reported a “dry” mouth showed worse oral health behaviors. The same results were found for the OHRQoL, which was lower when the number of remaining teeth was less than 20 (F = 3.384, *p* = 0.037) and when there subjective dry mouth was reported (F = 11.461, *p* < 0.001). However, there were no significant differences in these variables according to objective oral dryness among the oral health characteristics. 

### 3.4. Correlations of Oral Health Knowledge and Behavior and OHRQoL 

Oral health knowledge had a positive correlation with oral health behavior (r = 0.175, *p* = 0.029) and OHRQoL (r = 0.254, *p* = 0.003). Oral health behavior was positively correlated with OHRQoL (r = 0.239, *p* = 0.005). 

### 3.5. Factors Affecting OHRQoL 

Table 3 shows the results of the multiple regression analysis performed to identify the factors influencing quality of life related to oral health. The assumption of normality was confirmed through the normal P-P plot and histogram of the regression standardized residual. In addition, equal variance and independence were confirmed using a standardized residual scatter plot. Since the Durbin–Watson test value was 2.365, which is close to 2, it was confirmed that they are mutually independent, and the value of the variance inflation factor (VIF) was 1.09 to 2.98, indicating a value less than 10, which is suitable for regression analysis because there is no problem with polycollinearity.

For the regression model, the variables significantly associated with OHRQoL in the univariate analysis were selected; thus, age, education level, average monthly income, remaining teeth, subjective oral dryness, objective oral dryness, and oral health knowledge and behavior were the independent variables, and OHRQoL was the dependent variable. Subjective oral dryness was found to significantly influence OHRQoL, being the only significant predictor of OHRQoL (β = −4.995, *p* = 0.001), which explained 22.5% of the variance.

In contrast, the remaining variables included in the analysis were not significant factors. Thus, oral dryness as a symptom experienced by participants affected OHRQoL, while other factors that indirectly affect oral health, such as oral health knowledge and behavior, did not significantly affect OHRQoL.

## 4. Discussion

This descriptive correlational study analyzed the factors affecting OHRQoL; identified the differences in oral health knowledge, behavior, and OHRQoL according to general and oral health-related characteristics, and the correlations of the variables; and determined the degree of variables’ impact on OHRQoL.

The results of this study showed that a higher age was associated with lower oral health knowledge. This corresponds with the results of previous studies showing that younger age is associated with higher oral health knowledge [18]. The data also indicated that oral health behavior was lower for participants with a lower educational background and those with lower economic income. This is consistent with previous studies showing that older adults who are relatively young show better oral health behaviors [35]. In addition, previous studies showed that higher age, lower education, and lower income negatively affect OHRQoL [15], and these results were confirmed in this study. These results show that the elderly in rural areas are more vulnerable to poor oral health. People in rural areas are less educated, have lower income, and are older than those in urban areas [15]. In this study, the ratio of low-educated elderly (under middle school graduate) is about 60%, which is much higher than the ratio of low-educated elderly in Korea (23.53%). Moreover, 36.7% of all participants belonged to the low-income group, and had a monthly average income of 700,000 won or less. The number of participants aged 60–65 years in this study was large. This is because the current study only included participants who were able to fill out a questionnaire and cooperate with the measurement. Further research is needed to include a wider range of elderly age groups. Among the general characteristics, OHRQoL according to gender and chronic disease was not statistically significant. The results of gender are different for previous studies [35,36,37]; thus, further gender-based studies are needed. However, older adults use polypharmacy due to chronic diseases, and certain drugs also cause dry mouth [38,39]. Therefore, follow-up studies including these factors are required.

The results of this study showed that subjective oral dryness was a significant factor affecting OHRQoL. This indicates that oral dryness perceived by oneself is very important, which is in line with previous studies reporting that lower subjective oral dryness was associated with better OHRQoL [40,41,42]. In this study, an approved medical device indicated that 95.7% of the participants showed problems with salivary function, and the objective dryness of the oral cavity was very serious; however, only 36% of the participants experienced subjective dry mouth. Previous studies have shown that older people are not aware of subjective oral dryness symptoms until their salivation is severely reduced, i.e., by 50% or more, due to impaired oral sensory function [43,44]. The same results were found in the results of this study. The objective oral dryness for OHRQoL was not statistically significant. This is because even if there is an abnormality in the salivary function, the symptoms are not recognizable [43]. However, serious damage to the salivary function is difficult to recover from and must be prevented in advance [39]. Therefore, it is necessary to make a diagnosis using a device that can objectively measure salivation function before it is impaired. Based on these results, a comprehensive and holistic assessment of oral dryness is necessary. Particularly in rural areas, the role of public healthcare is more important due to the lack of information on education and health, and poor access to medical care [13].

As a result of the regression analysis, oral health knowledge and behavior did not significantly affect OHRQoL. In previous studies, correct oral health knowledge and proper oral health behaviors showed an effect on improving OHRQoL [20,21,45,46], which is in contrast to the results of this study; thus, further exploration is needed. However, a positive correlation was confirmed between oral health knowledge, oral health behavior, and OHRQoL, and these results are consistent with those of previous studies [47,48]. Oral health problems can be easily prevented through routine oral health management [17]. In oral health management, correct oral health knowledge and behavior is important [49]. In this way, oral health education is essential to promote correct oral health knowledge and behavior to improve OHRQoL. Older adults need oral health education that reflects their characteristics, as their knowledge and behavior about oral health is rigid and difficult to change [50]. However, current oral health education is limited to simple oral health knowledge provision or brushing education [51]. Therefore, healthcare providers should develop educational programs that provide detailed oral health knowledge that can be directly linked to oral health behavior; moreover, it is necessary to devise and implement not only one-time education, but also a plan for continuous oral health education. 

When evaluating oral health for older adults, healthcare providers should comprehensively and systematically assess oral dryness in terms of causes, such as checking their health condition, monitoring the use of drugs that cause oral dryness, and stopping unnecessary ones. In rural areas, the role of public medical institutions is even more important. At the frontline of the community, healthcare providers should raise awareness of the need for oral health and provide oral health education so that oral health problems can be prevented. In addition, oral health education methods and programs should be developed for continuing education to change oral health knowledge and behavior.

### Limitations

This study has some limitations due to the sample and methodology. First, the study used convenience sampling. The participants were biased toward 60–65-year-olds; thus, the study results may not be generalizable to all elderly people in rural areas. Second, the methodology restricted participation and may have impacted the results. Only participants who were able to complete the questionnaire and who were able to cooperate when measuring oral dryness were able to participate in this study. Finally, only 22.5% of the variance of OHRQoL could be explained. This raises the need to review more diverse variables together with the variables examined in this study. In particular, it seems meaningful to examine co-morbidity, drugs, cognitive function, and depression tendency together.

## 5. Conclusions

We confirmed that the increase in oral health knowledge and behavior also increases the OHRQoL. In addition, subjective oral dryness was found to be an important factor in the OHRQoL. However, when subjective oral dryness occurs, it is usually already after the salivary function has been impaired. Therefore, to prevent subjective oral dryness in advance, it is necessary to predict the salivary function impairment in the elderly through objective oral dryness measurement. In addition, rather than a simple approach to oral health problems, holistic and integrated interventions that evaluate the drugs being taken, systemic diseases, and psychological aspects are required. Development of prevention programs and continuous education that can improve oral health knowledge and behavior is crucial.

## Figures and Tables

**Table 1:** Demographic and oral health characteristics (*n* = 117).

Characteristics	Categories	*n*	%	M ± SD
Gender	Male	69	59.0	
	Female	48	41.0	
Age (years)	65~69	76	65.0	69.85 ± 5.21
	70–79	30	25.6	
	≥80	11	9.4	
Education level	≤Elementary school graduate	32	27.4	
	Middle school graduate	31	26.4	
	High school graduate	35	30.0	
	≥University graduate	19	16.2	
Marital status	Unmarried (single, divorce, etc.)	21	17.9	
	Married	96	82.1	
Monthly income (10,000 won)	<70	43	36.7	
	70–150	45	38.5	
	>150	29	24.8	
Present illness	Yes	103	88.0	
	No	14	12.0	
Oral health education experience	Yes	36	30.8	
	No	81	69.2	
Remaining teeth	<20	48	41.0	18.81 ± 8.97
	20–25	25	21.4	
	>25	44	34.2	
Subjective oral dryness	Dryness	43	36.8	
	Moderate	52	44.4	
	Never dry	22	18.8	
Objective oral dryness	≤27.9	90	76.9	26.12 ± 2.62
	28.0–29.5	22	18.8	
	≥29.6	5	4.3	

*n* = number; M = mean; SD = standard deviation.

**Table 2:** Oral health knowledge and behavior and oral health-related quality of life (OHRQoL), according to general and oral health characteristics (*n* = 117).

Characteristics	Categories	Oral Health Knowledge			Oral Health Behavior			OHRQoL		
		M ± SD	t or F (*p*)	Scheffe	M ± SD	t or F (*p*)	Scheffe	M ± SD	t or F (*p*)	Scheffe
Gender	Male	4.81 ± 1.69	1.163 (0.247)		16.55 ± 4.26	1.005 (0.317)		43.90 ± 11.85	1.604 (0.111)	
	Female	4.46 ± 1.50			15.77 ± 3.91			40.25 ± 12.43		
Age (years)	65–69 ^a^	4.96 ± 1.47	3.815 (0.025) *	a > b	17.18 ± 4.04	7.082 (0.001) **	a > b, c	45.13 ± 10.45	7.514 (0.001) **	a > c
	70–79 ^b^	4.07 ± 1.70			16.90 ± 3.58			39.20 ± 12.91		
	≥80 ^c^	4.27 ± 1.95			13.27 ± 4.00			32.27 ± 14.67		
Education level	≤Elementary school graduate ^a^	4.13 ± 1.81	1.804 (0.150)		13.09 ± 3.81	10.663 (<0.001) ***	a < b, c, d	35.44 ± 12.18	7.013 (<0.001) ***	a < c, d
	Middle school graduate ^b^	4.87 ± 1.69			17.35 ± 3.34			41.68 ± 11.09		
	High school graduate ^c^	4.77 ± 1.46			17.29 ± 3.95			47.34 ± 10.86		
	≥University graduate ^d^	5.05 ± 1.31			17.74 ± 3.54			46.21 ± 11.05		
Monthly income (10,000 won)	≤70 ^a^	4.42 ± 1.80	2.274 (0.107)		14.40 ± 4.17	7.493 (0.001) **	a < b, c	37.58 ± 12.87	5.752 (0.004) **	a < b, c
	70–150 ^b^	4.56 ± 1.56			17.22 ± 3.91			45.16 ± 10.92		
	≥150 ^c^	5.21 ± 1.32			17.41 ± 3.48			45.28 ± 11.05		
Present illness	Yes	4.73 ± 1.58	−1.116 (0.267)		16.25 ± 4.10	−0.153 (0.878)		42.21 ± 12.15	0.452 (0.652)	
	No	4.21 ± 1.89			16.07 ± 4.46			43.79 ± 12.69		
Oral health education experience	Yes	4.58 ± 1.68	−0.370 (0.712)		18.31 ± 3.70	3.832 (<0.001) ***		41.22 ± 12.95	−0.697 (0.487)	
	No	4.70 ± 1.60			15.31 ± 3.99			42.93 ± 11.85		
Remaining teeth	<20 ^a^	4.35 ± 1.76	1.639 (0.199)		15.06 ± 3.97	5.814 (0.004) **	a < c	39.40 ± 12.03	3.384 (0.037) *	a < c
	20–25 ^b^	4.77 ± 1.70			15.73 ± 4.50			42.15 ± 12.80		
	>25 ^c^	4.95 ± 1.34			17.84 ± 3.61			45.91 ± 11.26		
Subjective oral dryness	Dryness ^a^	4.37 ± 1.72	1.191 (0.309)		14.72 ± 4.05	5.463 (0.005) *	a < b, c	36.33 ± 12.34	11.461 (<0.001) ***	a < b, c
	Moderate ^b^	4.81 ± 1.66			16.79 ± 3.62			44.48 ± 10.72		
	Never dry ^c^	4.91 ± 1.23			17.86 ± 4.60			49.36 ± 9.89		
Objective oral dryness	≤27.9 (oral dryness)	4.64 ± 1.59	2.185 (0.117)		16.03 ± 4.31	0.533 (0.588)		42.24 ± 12.31	0.032 (0.968)	
	28.0–29.5	5.05 ± 1.70			16.73 ± 3.60			42.95 ± 12.78		
	≥29.6	3.40 ± 1.14			17.60 ± 2.88			42.80 ± 8.07		

*** *p* < 0.001; ** *p* < 0.01; * *p* < 0.05. ^a, b, c, d^: represents for each category.

**Table 3:** Results of multivariate regression of factors associated with OHRQoL (*n* = 117).

Variables	OHRQoL					
	Β	SE	β	T	*p*	95%CI
Oral Health Knowledge	1.261	420.639	0.168	1.964	0.052	−0.01 to 2.53
Oral Health Behavior	0.036	0.28	0.012	0.13	0.897	−0.52 to 0.59
General Characteristics						
Age (year)	−0.128	0.287	−0.055	−0.447	0.655	−0.70 to 0.44
Education Level (Ref. = ≥University Graduate)						
≤Elementary school graduate	−4.916	4.341	−0.181	−1.132	0.26	−13.52 to 3.69
Middle school graduate	−4.287	3.363	−0.156	−1.275	0.205	−10.96 to 2.38
High school graduate	0.76	3.342	0.029	0.228	0.82	−5.87 to 7.38
Monthly Income (10,000 won) (Ref. = ≥150)						
≤70	1.187	3.179	0.047	0.373	0.71	−5.12 to 7.49
70–150	2.902	2.81	0.092	0.819	0.414	−3.27 to 7.87
Oral health characteristics						
Remaining Teeth	0.169	0.134	0.125	1.266	0.208	−0.10 to 0.44
Subjective Oral Dryness	−4.995	1.529	−0.298	−3.267	0.001 *	−0.94 to 0.63
Objective Oral Dryness	−0.152	0.397	−0.033	−0.384	0.702	1.96 to 8.03
	Adj R^2^ = 0.225, F (*p*) = 4.057 (<0.001)					

B = regression coefficient; SE = standard error of the regression coefficient; CI = confidence interval; * *p* < 0.05.

## Data Availability

The data are not publicly available because of privacy or ethical restrictions.
